# Mechanical Characteristics, In Vitro Degradation, Cytotoxicity, and Antibacterial Evaluation of Zn-4.0Ag Alloy as a Biodegradable Material

**DOI:** 10.3390/ijms19030755

**Published:** 2018-03-07

**Authors:** Ping Li, Christine Schille, Ernst Schweizer, Frank Rupp, Alexander Heiss, Claudia Legner, Ulrich E. Klotz, Jürgen Geis-Gerstorfer, Lutz Scheideler

**Affiliations:** 1 Section Medical Materials Science and Technology, University Hospital Tübingen, Osianderstrasse 2–8, 72076 Tübingen, Germany; pingli1015@gmail.com (P.L.); Christine.Schille@med.uni-tuebingen.de (C.S.); Ernst.Schweizer@med.uni-tuebingen.de (E.S.); Frank.Rupp@med.uni-tuebingen.de (F.R.); Juergen.Geis-Gerstorfer@med.uni-tuebingen.de (J.G.-G.); 2 Research Institute for Precious Metals and Metals Chemistry (fem), Katharinenstrasse 17, 73525 Schwäbisch Gmünd, Germany; heiss@fem-online.de (A.H.); legner@fem-online.de (C.L.); klotz@fem-online.de (U.E.K.)

**Keywords:** biomaterials, biodegradable metals, zinc alloy, corrosion, cytotoxicity

## Abstract

Zn-based biodegradable metallic materials have been regarded as new potential biomaterials for use as biodegradable implants, mainly because of the ideal degradation rate compared with those of Mg-based alloys and Fe-based alloys. In this study, we developed and investigated a novel Zn-4 wt % Ag alloy as a potential biodegradable metal. A thermomechanical treatment was applied to refine the microstructure and, consequently, to improve the mechanical properties, compared to pure Zn. The yield strength (YS), ultimate tensile strength (UTS) and elongation of the Zn-4Ag alloy are 157 MPa, 261 MPa, and 37%, respectively. The corrosion rate of Zn-4Ag calculated from released Zn ions in DMEM extracts is approximately 0.75 ± 0.16 μg cm^–2^ day^–1^, which is higher than that of pure Zn. In vitro cytotoxicity tests showed that the Zn-4Ag alloy exhibits acceptable toxicity to L929 and Saos-2 cells, and could effectively inhibit initial bacteria adhesion. This study shows that the Zn-4Ag exhibits excellent mechanical properties, predictable degradation behavior, acceptable biocompatibility, and effective antibacterial properties, which make it a candidate biodegradable material.

## 1. Introduction

Biodegradable metals (BMs) are regarded as the next revolutionary metallic biomaterials and have become a potential alternative to permanent biomaterials during the last decade [1,2,3]. Magnesium, iron, zinc, and their related alloys, have been intensively investigated for their potential as BMs. However, the rapid corrosion rate accompanied by the accumulation of hydrogen in physiological environments impedes the clinical application of Mg-based alloys [4,5]. Fe-based alloys, on the contrary, exhibit relatively slow degradation rates and excellent mechanical properties, but superior corrosion resistance may impede the desired replacement by newly-formed tissue [6,7].

In comparison with Mg and Fe, the standard corrosion potential of Zn (−0.762 V_SCE_) is between Fe (−0.440 V_SCE_) and Mg (−2.372 V_SCE_) [1,2,8]. Bowen et al. [9] reported the biocompatibility and degradation of zinc wires implanted into the abdominal aorta of rats, and zinc wires exhibited moderate degradation rates in vivo for up to 6.5 months. Moreover, zinc is one of the essential nutrients in the human body, where it influences various normal physiological processes [10,11]. Additionally, considering bio-safety, the recommended allowances for elemental zinc are estimated at 15 mg day^–1^ [1]. In addition to its excellent corrosion and biocompatibility properties, Zn is also one of only a few metals with high magnetic resonance imaging compatibility, which is superior to that of Mg alloys and Fe alloys. The magnetic (volume) susceptibility of Zn, Mg, and Fe are −15.7 × 10^6^, +11.7 × 10^6^, and +0.2 × 10^6^, respectively [12]. Therefore, these advantages make Zn-based alloys promising candidates for a new generation of BMs, especially for use as osteosynthesis materials and cardiovascular stents [9,13].

**Figure 1:**
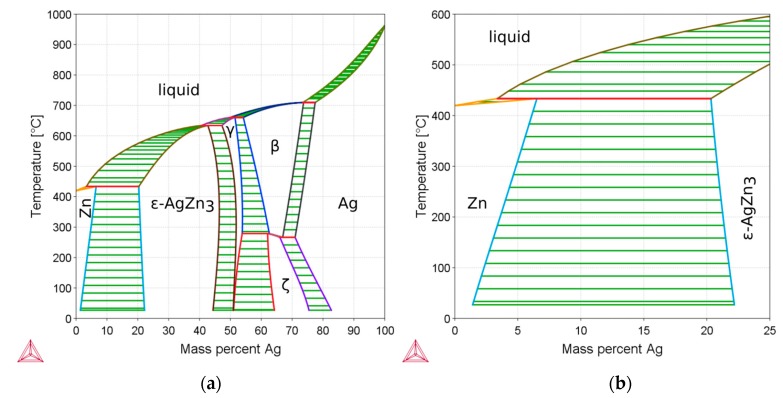
(**a**) Calculated Zn-Ag phase diagram using the Themo-Calc 2017a software (Thermo-Calc Software AB, Solna, Sweden) and the SNOB-3 database. (**b**) Detail of the phase diagram in (**a**) manifesting that up to 6 wt % Ag can be solved in Zn. Upon cooling, the composition enters the two-phase area, i.e., precipitations of ε-AgZn_3_ in the Zn matrix occur. As this effect is generally accompanied by an increase in strength, it is referred to as precipitation hardening.

Regarding the clinical requirements, the application of pure Zn in BMs is limited because of its weak strength, plasticity, and low hardness. It has been investigated that the tensile strength of pure Zn is from 10 MPa to 110 MPa, the elongation is 0.32% to 36%, and the Vickers hardness is 38 HV1 to 39 HV1, being insufficient mechanical properties for most clinical applications [13,14]. Thus, biodegradable Zn-based alloys with superior mechanical properties should be developed to meet the clinical requirements. Improvements in mechanical properties may be achieved by adding alloying elements and/or appropriate thermomechanical treatment, such as extrusion, rolling, forging, annealing, and so forth [15,16]. In BMs, the biocompatibility of alloying elements must be carefully considered. In this work, Ag is proposed as an alloying element in Zn-based alloys, since it can improve mechanical properties. According to the phase diagram (Figure 1), up to 6 wt % Ag is solvable in Zn at temperatures of about 400 °C. As the solubility decreases upon cooling, ε-AgZn_3_ precipitates form. Thus, dislocations are pinned by the precipitates resulting in improved hardness and strength (precipitation hardening). Zn-Ag binary alloys have been investigated and Ag has been proven to improve the mechanical properties efficiently [17]. Moreover, the Ag ion shows antibacterial functions and has already been used as an alloying element [18]. Adding Ag has shown promising antibacterial properties in Mg-based alloys while preserving the biocompatibility [18].

In this study, the aim was to develop and investigate a Zn-4 wt % Ag alloy as a novel biodegradable metal. The Zn-4Ag alloy was prepared, and thermomechanical treatment was applied to refine the microstructure and improve the mechanical properties. The microstructure, mechanical properties, and corrosion behavior of the Zn-4Ag alloy were investigated. Furthermore, the cytotoxicity and antibacterial properties were also evaluated.

## 2. Materials and Methods

### 2.1. Materials Preparation

Alloys were prepared from high purity elements (>99.9%) by induction melting (Indutherm VC 500 D; Indutherm GmbH, Walzbachtal, Germany) under 1 bar Argon in a graphite crucible. An oxide scavenger (Zincrex D85; Feuerungsbau Mutschler GmbH, Neckartenzlingen, Germany) was employed to clear the melt. The melt (750 °C) was cast into a cylindrical graphite mold 15 mm in diameter. During solidification, the mold was vibrated, resulting in a reduction in grain size from approximately 200 µm to about one tenth this size.

All casting rods were homogenized at 300 °C for 1 h in a furnace under Ar protective gas and then left in the furnace for cooling. The moderate cooling rate allowed phase separation and grain growth, which proved to be advantageous for the subsequent hot working. The rods were first machined to a diameter of 10 mm and then swaged to 3 mm diameter wires. As the rods proved to be too brittle for swaging at room temperature, rods and tool were preheated to 200 °C. Subsequently, the wires were annealed at 390 °C for 15 min, quenched in water and, finally, precipitation hardened in an oil bath for 10 min at 100 °C. An inductively-coupled plasma optical emission spectrometry (ICP-OES) analysis of the alloy confirmed its composition.

For the corrosion tests and the biological tests, small plates with a dimension of 7 mm × 7 mm × 0.5 mm were prepared analogously to the wires by casting into a rectangular graphite mold with a thickness of 10 mm, homogenization at 300 °C for 1 h, hot rolling at 200 °C, annealing at 390 °C for 15 min and, finally, cutting. Samples were ground with P1200 SIC paper (Buehler-Wirtz GmbH, Düsseldorf, Germany) using a grinding machine (Meta Serv; Buehler) and ultrasonically cleaned (Sonorex super RK102H; Bandelin electronic GmbH & Co. KG, Berlin, Germany) with absolute ethanol for 10 min. Each side of the specimens was further sterilized by ultraviolet radiation for at least 1 h in a sterile workbench (Lamin Air HB2472; Heraeus, Hanau, Germany) for corrosion testing, cytotoxicity, and antibacterial evaluation.

### 2.2. Microstructure Observation and Mechanical Characteristic Test

Metallographic cross-sections of each processing step were prepared, etched with 2% Nital, a mixture of EtOH and HNO_3_, and routinely subjected to a light microscopic investigation (Zeiss Axioplan 2; Carl Zeiss Microscopy GmbH, Oberkochen, Germany). Vickers hardness (diamond pyramid hardness), here denoted as HV1, was measured on metallographically-polished cross-sections using a load of 1 kg. Hardness is generally proportional to ultimate tensile strength (UTS) values, but it does not provide information about the ductility of an alloy. Wires 3 mm in diameter were subjected to tensile testing according to DIN EN ISO 10002-1 in a Zwick Z100HT universal testing machine (Zwick GmbH & Co. KG, Ulm, Germany) at room temperature. The strain was measured until fracture using a strain gauge on a starting length of 15 mm. The testing speed was 1.5 mm/min until the yield strength was surpassed and then increased to a strain controlled strain rate of 0.0025 s^−1^. The values for 0.2% yield strength (YS_0.2_), ultimate tensile strength (UTS), and elongation (ε_f_) were determined.

Prior to an investigation in the scanning electron microscope (SEM), all cross-sections were subjected to a broad argon ion beam polishing procedure (BIB, sample rotation, 3° incident angle, 6 kV, 2.2 mA, 15 cycles: 2 min beam on, 15 min rest) using a Bal-Tec RES 101 (now Leica Microsystems GmbH, Wetzlar, Germany). The SEM investigations were conducted with a Zeiss Auriga 60 (Carl Zeiss Microscopy GmbH, Oberkochen, Germany) equipped with a field emission gun and an 80 mm^2^ SDD EDX-Detector (X-Max 80, Oxford Instruments, Abingdon-on-Thames, UK).

A Bruker D8 GADDS diffractometer (Bruker AXS GmbH, Karlsruhe, Germany) in GADDS configuration (“General Area Detector Diffraction System”) equipped with a Våntec-500 2D detector (Bruker AXS GmbH) was employed for X-ray diffraction (XRD) based phase analysis. The X-ray beam (λ(Cu Kα) = 1.54 Å) was adjusted using a Göbel mirror and a 1 mm collimator. Acquired diffraction rings were integrated to one-dimensional diffraction pattern using the GADDS v4.5 and MERGE v2 software packages (Bruker AXS GmbH). Phase content was evaluated usingthe software DIFFRAC.EVA v2 (Bruker AXS GmbH) and the database (ICDD-PDF-2).

### 2.3. Extract Preparation

The extracts of Zn-4Ag alloy and pure Zn were prepared in DMEM (Dulbecco’s modified Eagle medium; Gibco DMEM, Thermo Fisher Scientific GmbH, Karlsruhe, Germany) containing 10% fetal calf serum (FCS; PAA Laboratories GmbH, Cölbe, Germany), 1% 200 mM l-glutamine (PAA), and 1% penicillin 10 mg/mL (Gibco, Thermo Fisher Scientific GmbH, Karlsruhe, Germany) and McCoy’s 5A (Sigma-Aldrich Chemie GmbH, Steinheim, Germany) supplemented with 15% FCS, 1% 200 mM l-glutamine and 1% penicillin 10 mg/mL at 37 °C in 5% CO_2_ for 24 h. The ratio of surface area (cm^2^) to solution volume (mL) was set to 3 cm^2^ mL^–1^ for all samples, according to ISO 10993-12:2012 [19]. Thereafter, the extracts were diluted to four testing concentrations, namely 10% extracts (dilution factor 1:10), 16.7% extracts (dilution factor 1:6), 33.3% extracts (dilution factor 1:3) and 100% extracts, according to the recommendation in [20]. The extracts were further used for corrosion rate determination and cytotoxicity evaluation.

### 2.4. Corrosion Rate Determination

The estimated corrosion rates were calculated from released ions in the extracts according to the previous studies [21,22,23]. An ICP-OES (Optima 4300 DV, Perkin Elmer, Rodgau, Germany) was employed to detect released Zn and Ag ions in the extracts. Triple diluted extracts (1:3) were used for the measurements. Released Zn and Ag ions were measured at two different wavelengths with three-time repetition. The corrosion rate was calculated from released Zn ions using the following formula, according to [24]:

Corrosion rate (μg cm^−2^ day^−1^) = (C × V)/(S × T) (1)

where C is the released Zn ion concentration in μg/mL, V is the solution volume in mL, S is the sample surface area in cm^2^, and T is the incubation time in days. The surface morphology and chemical composition of the corrosion products on the surfaces after immersion were also observed using a scanning electron microscope equipped with an energy dispersive X-ray spectrometer (SEM-EDX; LEO 1430, Carl Zeiss GmbH, Oberkochen, Germany).

### 2.5. Cytotoxicity Tests

The cytotoxicity evaluation of Zn-4Ag was performed via extract test, according to ISO 10993–5: 2009 [25]. L929 fibroblasts (mouse fibroblast cell line, DSMZ GmbH, Braunschweig, Germany) and Saos-2 osteoblasts (Human primary osteosarcoma cell line, DSMZ GmbH, Braunschweig, Germany) were used. Cytotoxicity was tested for two biological endpoints: metabolic activity (Roche Cell Proliferation Kit II, XTT assay; Roche Diagnostics GmbH, Mannheim, Germany) and cell proliferation (Roche cell proliferation ELISA, BrdU assay). Ti-6Al-4V alloy (Camlog GmbH, Wimsheim, Germany) was used as negative control. L929 fibroblasts were cultured in 24 mL DMEM medium and Saos-2 osteoblasts were cultured in 10 mL McCoy’s 5A, supplemented as described above. Both cell types were grown in 75 cm^2^ culture flasks (Costar, Merck KGaA, Darmstadt, Germany) at 37 °C in a humidified atmosphere with 5% CO_2_.

For the tests, L929 fibroblasts and Saos-2 osteoblasts were seeded in 96-well plates (200 μL/well) at a cell density of 1 × 10^4^ cells per well and pre-incubated overnight. Thereafter, 150 μL of the respective extract dilutions replaced the cell medium (four parallel wells per dilution). After 24 h incubation with these extracts, 50 μL XTT reagent was added to each well for 2 h. Subsequently, the formazan formation was determined photometrically using an ELISA reader (Biotek, Bad Friedrichshall, Germany) at the wavelengths of 450/620 nm. Proliferative activity of L929 and Saos-2 was determined in the logarithmic growth phase between 24 h and 48 h after seeding by BrdU assay. Fifteen microliters of BrdU labeling reagent were added to each well 24 h after seeding. Additional cell cultures without BrdU-label were used as background controls. Culture medium without cells containing BrdU and Anti-BrdU-POD was used as blank controls. Forty-eight hours after seeding, the cells were fixed, and Anti-BrdU-POD was added according to manufacturer’s instructions. The absorbance of the samples was measured using an ELISA Reader at 450/690 nm.

### 2.6. Antibacterial Effect Evaluation

For determining bacterial adhesion, Zn-4Ag samples were inoculated with *Streptococcus gordonii* strain DL1 (*S. gordonii*) and adhering bacteria were determined using a crystal violet staining assay (0.5% crystal violet in 20% methanol) and a fluorescent nucleic acid stain (Live/Dead BacLight Bacterial Viability Kit, Invitrogen L13152, Thermo Fisher Scientific GmbH, Karlsruhe, Germany). Bacteria were grown as a stationary suspension culture in Schaedler medium (Beckton Dickinson GmbH, Heidelberg, Germany) overnight at 37 °C. Thereafter, 4 mL *S. gordonii* suspension were added to each sample in six-well plates and cultivated for 12 h at 37 °C. After incubation for 12 h, the *S. gordonii* suspension was carefully removed and samples were immersed in 3 mL crystal violet solution for 20 min. After staining, the samples were rinsed three times with deionized water. Subsequently, the samples were observed and photo-documented under a photomacroscope (Wild M 400, Wild, Heerbrugg, Switzerland) equipped with a remote control DSLR (Nikon 550D, Nikon, Tokyo, Japan). For live/dead staining, the samples were rinsed two times with Hanks’ salt solution (Biochrom AG, Berlin, Germany). Live/dead staining was used to evaluate the live/dead state of bacteria on the surface, following manufacturer’s instructions. Biofilm formation and adherent bacteria were examined with a fluorescence microscope (Optiphot-2, Nikon, Tokyo, Japan) equipped with a remotely controlled DSLR. Ti-6Al-4V samples were selected as a reference.

### 2.7. Statistical Methods

The inhibition of metabolic activity of the cells (XTT) was determined in three independent experiments, and the proliferation tests (BrdU-incorporation) were performed twice. The combined results of the respective cytotoxicity tests are given as mean values ± standard deviation. Statistical significance of differences between groups was tested by Student’s *t*-test. Differences of *p*-values < 0.05 were considered statistically significant.

## 3. Results and Discussion

### 3.1. Microstructure and Mechanical Properties

**Figure 2:**
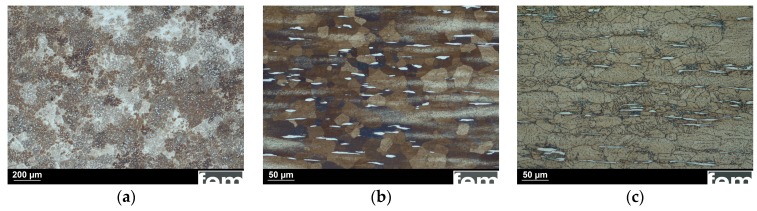
Optical micrograph of: (**a**) the as-cast Zn-4Ag dendritic microstructure; (**b**) longitudinal sections after thermomechanical treatment (homogenization, swaging and solution annealing); and (**c**) after precipitation hardening showing globular grains. Large ε-AgZn_3_ grains (bright) can be identified in the cross-sections.

The evolution of the microstructure was investigated by light and scanning electron microscopy. A heat treatment (homogenization, 300 °C for 1 h) induced a transformation of the dendritic as-cast microstructure (Figure 2a) to large globular grains. After the thermomechanical treatment, i.e., swaging, annealing (390 °C for 15 min), and quenching, bright grains corresponding to the ε-AgZn_3_ phase are visible (Figure 2b). Subsequent precipitation hardening (100 °C for 10 min) led only to slightly larger grains, but did not further affect the microstructure (Figure 2c).

Concerning thermomechanical treatment and precipitation hardening, the resulting mechanical properties are compiled in Table 1. A minor modification of the Mg alloy WE43 is already used for bioresorbable stents. The material exhibits reasonable mechanical properties, but the corrosion rate is high, a controversial issue among experts [26]. Bowen et al. [27] investigated the qualification of metallic zinc for bioresorbable stents. They concluded that the comparably slow corrosion rate and the low toxicity of the resulting products make zinc a promising candidate for bioresorbable stents. However, the authors acknowledge the insufficient mechanical properties of pure zinc, excluding a straight-forward application, and discuss putative additives. A recent publication shows that the addition of Ag results in considerably improved mechanical properties [17], which is in good agreement with the data presented here. Zn-4Ag shows good mechanical properties. Subsequent precipitation hardening (100 °C for 10 min) did not improve the mechanical properties. While the hardness slightly increases, YS und UTS both slightly decrease. This minor loss of strength is probably due to grain growth. Nevertheless, a sufficiently large window between YS (157 MPa) and UTS (261 MPa), as well as an elongation of 37% represents an excellent starting point for future material developments.

**Figure 3:**
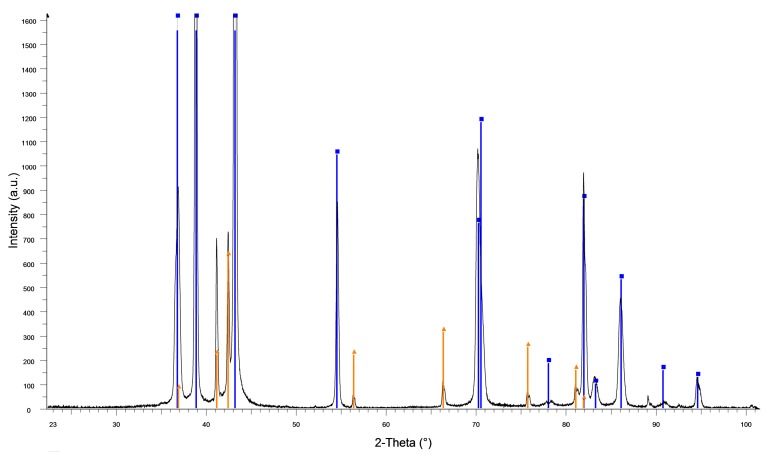
X-ray diffraction (XRD) pattern (black) of Zn-4Ag after thermomechanical treatment. The phases Zn (03–065–3358, blue) and ε-AgZn_3_ (00–025–1325, orange) were identified.

The missing aging effect of this heat treatment might be explained by the fact that the precipitates predominantly had formed beforehand. XRD analysis of the thermomechanically-treated state confirmed the presence of the two expected phases, Zn and ε-AgZn_3_ (Figure 3).

**Figure 4:**
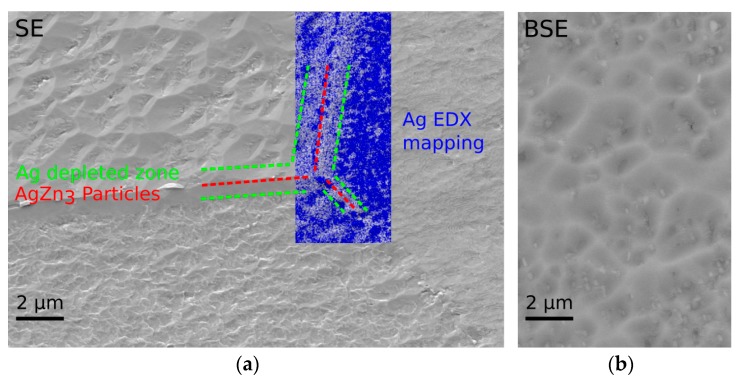
Scanning electron microscope (SEM) investigation of the microstructure after thermomechanical treatment. (**a**) For the sake of resolution, SE imaging of the BIB polished surface and the corresponding EDX analysis were performed at 6 kV. The overlay shows that ε-AgZn_3_ particles have formed along the grain boundaries while the proximity is Ag depleted; (**b**) the 20 kV BSE imaging revealed the presence of ε-AgZn_3_ precipitates within the Zn grains.

A micrograph acquired by surface sensitive secondary electron (SE) imaging at an accelerating voltage of 6 kV is shown in Figure 4a. Despite the gentle BIB polishing procedure, the surface is characterized by a certain grain orientation-dependent topography and surface roughness, respectively. Submicron sized, Ag-enriched ε-AgZn_3_ particles were identified along the Zn grain boundaries by EDX mapping (Figure 4a, blue colouring). Nucleation and growth of the ε-AgZn_3_ particles led on the other hand to a Ag depleted zone. Moreover, small precipitates inside the Zn grains were detected by backscatter electron imaging (BSE) at 20 kV (Figure 4b).

### 3.2. Corrosion Properties

**Figure 5:**
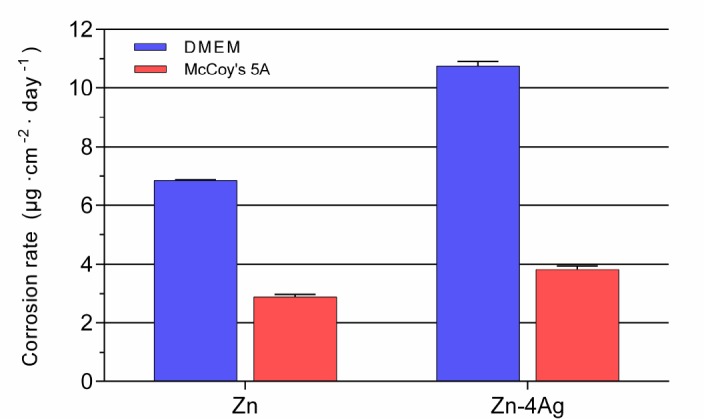
Corrosion rates of pure Zn and Zn-4Ag in DMEM and McCoy’s 5A calculated from released Zn ions.

In this study, corrosion rates of Zn-4Ag alloy and pure Zn were calculated from released Zn ions in the cell medium extracts (Figure 5), which correspond to the cytotoxicity of the Zn-4Ag alloy and pure Zn. The cell media used, DMEM and McCoy’s 5A, consist both of inorganic ions and organic components with concentrations equal to those in human plasma, as shown in Table 2. The use of extraction media with concentrations of buffering agents and glucose similar to those of human plasma is critical for predicting in vivo corrosion rates [30]. In these tests, using cell culture medium instead of simple salt solutions, the extract conditions are more closely related to the physiological environment in the body, although extraction time is only for 24 h. However, in vitro corrosion rates from long-term immersion tests in simulated body fluid should be further investigated.

As shown in Figure 5, corrosion rates of Zn-4Ag in DMEM and McCoy’s 5A were 10.75 ± 0.16 μg cm^–2^ day^–1^ and 3.80 ± 0.14 μg cm^−2^ day^−1^, respectively, which is higher than the counterpart of pure Zn, 6.85 ± 0.02 μg cm^–2^ day^–1^ and 2.89 ± 0.08 μg cm^–2^ day^–1^. It is clearly evident that higher corrosion rates in DMEM in comparison to McCoy’s 5A were also observed. The difference can be ascribed to the different composition of DMEM and McCoy’s 5A. One reason could be the higher (15%) concentration of FCS, which was used in McCoy’s 5A. It is known that the proteins in FCS can decrease corrosion rates, which was also observed in the case of Mg alloys [33].

In most previous studies, in vivo corrosion rates of Zn alloys were estimated by in vitro long-term immersion tests and electrochemical tests [2,31]. However, standardized in vitro methods for the corrosion rate determination that can mimic the degradation behavior of Zn alloys in the complex body physiologic environment are still lacking. In this study, the in vitro corrosion rate determination of Zn alloys was estimated from released Zn ions in DMEM for 24 h, as reported in previous studies [21,22,23]. Kubásek et al. [21] reported the corrosion rate of Zn-0.8Mg in DMEM was 13.4 ± 0.3 μg cm^–2^ day^–1^. This value is close to the calculated value of 10.75 ± 0.16 μg cm^–2^ day^–1^ in our present study. Jablonská et al. [22] reported a corrosion rate of Zn-1.5Mg calculated by released ions in extracts of 52 ± 10 μg cm^−2^ day^−1^ for an untreated control under a CO_2_ atmosphere, which is higher than the corrosion rate of Zn-4Ag in our study. The difference could be attributed to a different surface to volume ratio in the two studies and the fact that in the study of Jablonská et al. the solid corrosion products were dissolved by addition of ultrapure HNO_3_ prior to determination of the released ions.

In our study, adding Ag as alloying element to pure-Zn clearly increased the corrosion rate of the Zn-Ag binary alloy, which is consistent with previous findings [17]. Sikora-Jasinska et al. [17] reported that the corrosion rates of Zn-2.5Ag, Zn-5.0Ag, and Zn-7.0Ag alloys are from 79 to 84 μm year^−1^ in Hanks’ modified solution, which is higher in comparison to pure Zn. This might be related to the formation of the ε-AgZn_3_ phase, inducing micro-galvanic corrosion, which finally leads to a decreased corrosion resistance of Zn-Ag alloys. 

**Figure 6:**
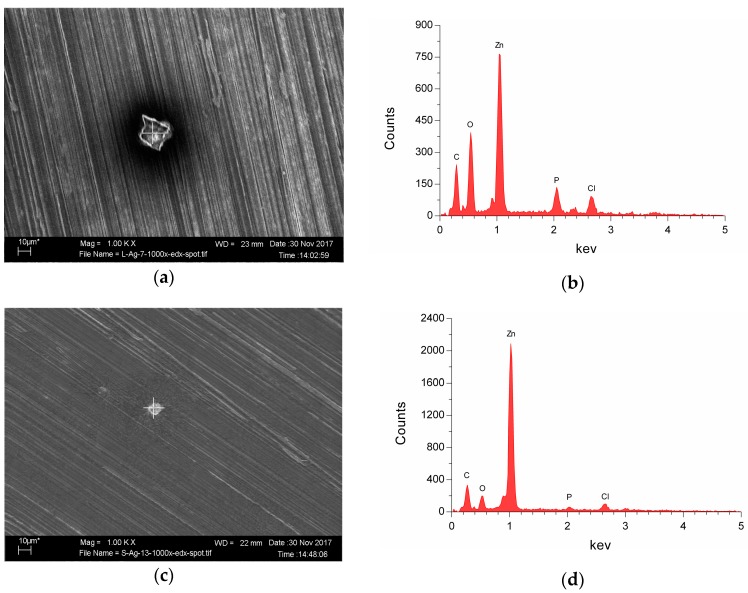
The SEM-EDX analysis of the Zn-4Ag alloy after immersion in DMEM/McCoy’s 5A for 24 h: (**a**) SEM images of Zn-4Ag alloy in DMEM (magnification 1000×); (**b**) EDX result of the degradation products in (**a**); (**c**) SEM images of Zn-4Ag alloy in McCoy’s 5A (magnification 1000×); and (**d**) EDX result of the degradation products in (**c**).

Figure 6 shows the surface morphologies detected by SEM and the EDX analyses of corrosion products on the surface of the Zn-Ag alloy after immersion for 24 h. The degradation products of Zn-4Ag were mainly similar as those of pure Zn. Only a small amount of white degradation products were distributed on the surface, and round particles formed on the surface were observed at high magnification (Figure 6a,c), which is consistent with previous studies [17,34]. The EDX analysis (Figure 6b,d) shows that these degradation products of Zn-4Ag alloy were mainly composed of Zn, O, P, C, and Cl, suggesting that these particles could be mainly composed of hydroxides, phosphate, carbonate compounds, and chloride salt formation, as reported in previous studies [13,16,35].

### 3.3. Cytocompatibility

**Figure 7:**
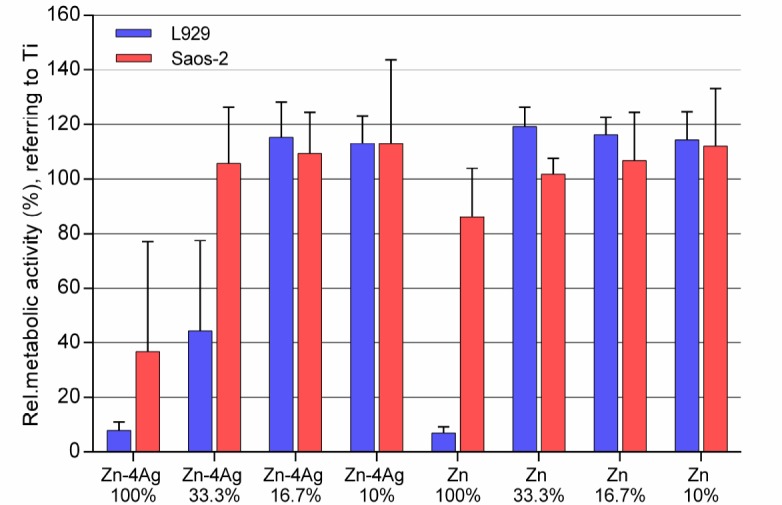
Effect of different concentrations of Zn-4Ag alloy and pure Zn extracts on the cell metabolic activity of L929 and Saos-2 determined by XTT assay. Ti-6Al-4V alloy was used as the negative control and was set to 100%. Means of three independent experiments are shown with respective standard deviations.

**Figure 8:**
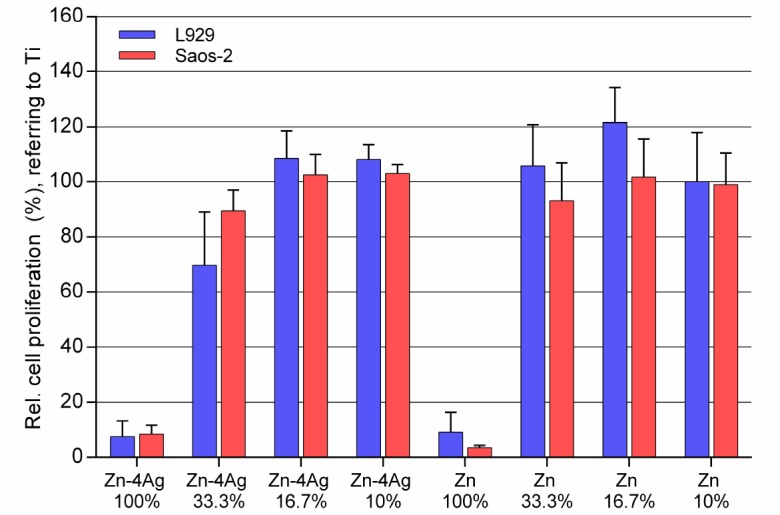
Influence of different concentrations of Zn-4Ag alloy and pure Zn extracts on the cell proliferation of L929 and Saos-2 determined by BrdU assay. Ti-6Al-4V alloy was used as negative control and was set to 100%. Means of two independent experiments are shown with respective standard deviations.

Table 3 shows the mean Zn ion concentration after incubation of pure Zn and Zn-4Ag alloy in DMEM/McCoy’s 5A to correlate the ion concentrations to the results of the cytotoxicity tests (Figure 7 and Figure 8). Analysis of the mean Zn ion concentration revealed that the highest concentration was determined for the Zn-4Ag alloy 100% extract in DMEM with 493.4 μM. Furthermore, the mean Zn ion concentration in DMEM extracts is higher than the counterpart in McCoy’s 5A. In addition, the mean Ag ion concentration in Zn-4Ag extracts was below the detection limit of the instrument, indicating a very limited release of Ag into the cell medium, although some Ag ions may be bound in the corrosion products.

Figure 7 shows the metabolic activity of L929 and Saos-2, respectively, cultured in 100%, 33.3%, 16.7%, and 10% extracts of Zn-4Ag for 24 h; pure Zn served as a control. According to ISO 10993-5: 2009, a decrease of cell viability higher than 30% is considered as a toxic effect. For the Zn-4Ag alloy, L929 cells and Saos-2 cells cultured in 100% Zn-4Ag extracts showed a much lower metabolic activity, below 40% of the control. Thus, these undiluted extracts showed a clear cytotoxicity. In the 33% Zn-4Ag extract, the cell viability of Saos-2 already approached 100%. L929 cells, on the contrary, reached approximately 40%. For 10% and 16.7% diluted extracts of Zn-4Ag, the metabolic activities of L929 cells and Saos-2 cells always reached 100%, and there was no statistically significant difference between Zn and Zn-4Ag groups (*p* > 0.05). Therefore, the Zn-4Ag alloy tested showed a certain degree of toxicity for L929 cells and Saos-2 cells based on the cytotoxicity results in our test systems.

Figure 8 shows the cell proliferation of L929 cells and Saos-2 cells cultured in 100%, 33.3%, 16.7%, and 10% extracts of Zn-4Ag and pure-Zn for 24 h determined by BrdU incorporation. In vitro cytotoxicity can be easily evaluated with tetrazolium salt-based assays. However, XTT assay and Cell Counting Kit-8 (CCK-8; Dojindo Molecular Technologies, Kumamoto, Japan) in our preliminary experiments were influenced by the degradation products of Zn-based alloys, for reasons which are probably similar to Mg-based alloys [36]. In contrast to the tetrazolium salt-based XTT-assay, the BrdU incorporation is a direct measure for proliferative activity. An additional advantage is that the BrdU assay is not prone to the interference of the released Zn ions with tetrazolium-based assays [36]. In 100% extracts of Zn-4Ag alloy and pure Zn, both cell types showed a total inhibition at proliferation ability, which was different compared to the results of the metabolic activity test. In the various diluted extracts of Zn-4Ag alloy and pure Zn, the proliferation activities of L929 and Saos-2 exhibited similar results as determined for the metabolic activities.

As a potential biodegradable material, the biocompatibility of the degradable Zn-4Ag alloy should be considered. In fact, the significance of Zn in human nutrition has been widely acknowledged. Zn is the second most abundant transition metal in the human body and an essential element for numerous biological functions [37,38]. The recommended intake for an adult is estimated at 15 mg day^−1^ [1,2]. In contrast, the human tolerance level for silver is estimated from 0.4 μg day^−1^ to 27 μg day^−1^ [39]. The effects evaluated by the extract test for the biodegradable Zn-based alloy are mainly attributed to the released corrosion products during the degradation process. In this study, a considerable released Zn ion concentration in extracts was found, while the Ag ion concentration was below the detection limit, indicating that the Zn ion concentration mainly determined the toxicity of the Zn-4Ag alloy. As far as systemic toxicity is concerned, the daily released Zn ions of the Zn-4Ag alloy are far below the above-allowed value. With the Zn ion concentration decreasing in the diluted extracts, the cytotoxic effect also decreased, demonstrating a concentration-dependent effect, consistent with several studies [40,41]. It is worth noting that significant differences in Zn ion concentrations were determined between both cell media under the same extraction conditions, which is obviously related to the different composition of the media.

Concerning cell sensitivity to zinc, Kubásek et al. [21] reported the Zn^2+^ safety concentrations for L929 and U-2 OS cells being 80 μM and 120 μM, respectively. In this present work, results similar to the above study were found. The mean Zn ion concentrations in 100% Zn-4Ag extract of DMEM and McCoy’s 5A were 493.4 μM and 174.4 μM, respectively. These values are higher than the safety concentrations, which explains the cytotoxic effects found in our tests. In our study, metabolic activity, determined by XTT assay, was less decreased by cytotoxic effects of the experimental alloy than proliferation determined by BrdU assay. This may be caused by the fact that metabolic activity of the cells is less sensitive to the cytotoxic action of the released Zn ions. However, interference of these ions with tetrazolium salts in the XTT assay may contribute to this effect.

For extract tests, most studies have found that only diluted extracts of biodegradable Zn-based alloys exhibited good cell viability [8,16,21], although even undiluted extracts have shown no cell toxicity in few studies [15,42]. The difference observed might be caused by different cell lines and different experimental Zn-based alloys. It has already been discussed that the current ISO 10993 standards for in vitro cytotoxicity tests (10993-5: 2009 and 10993-12: 2012) have only limited value for the evaluation of biodegradable metallic materials [19,25]. Wang et al. [20] suggested that a maximal ten-fold dilution (10% extracts) to a minimal six-fold dilution (16.7% extracts) of extracts for in vitro cytotoxicity tests were recommended for screening potential Mg-based alloys. According to this suggestion, the Zn-4Ag alloy in this study would have no potential cytotoxicity, according to the results of the 10% and 16.7% extracts. However, it is critical to perform in vivo systematic toxicity evaluation in future studies.

### 3.4. Antibacterial Evaluation

**Figure 9:**
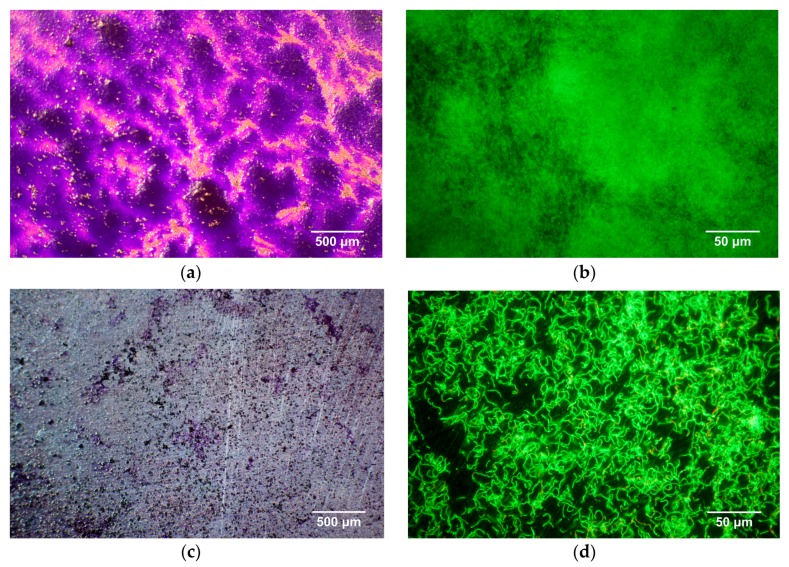
Biofilm formation and initial bacterial adhesion on Ti-6Al-4V alloy (**a**,**b**); and Zn-4Ag alloy (**c**,**d**) after incubation with *S. gordonii* for 12 h. Images obtained by: (**a**,**c**) crystal violet staining (magnification 32×); and (**b**,**d**) live/dead staining (magnification 400×).

The antibacterial properties of Zn-4Ag alloy to inhibit biofilm formation and initial bacterial adhesion was evaluated by crystal violet staining and live/dead staining, as shown in Figure 9. After 12 h incubation with *S. gordonii*, the surface of the Ti-6Al-4V alloy showed an intense violet staining and green fluorescence (Figure 9a,b), respectively, which means a high level of biofilm formation and a significant amount of adherent *S. gordonii*. In comparison, the Zn-4Ag alloy presented point-like violet staining and a comparatively thin layer of green fluorescent vital bacterial chains (Figure 9c,d), indicating the inhibition of initial *S. gordonii* adhesion and less biofilm formation compared with the Ti-6Al-4V alloy.

Postoperative infection is a common complication in surgical implants, and the infection rate ranges from 1% to 4.5% for dental implant surgery [43]. The postoperative infection not only leads to implant failure, but also delays tissue remodeling. In the present work, the Zn-4Ag alloy could effectively inhibit bacterial adhesion and biofilm formation in comparison to Ti-6Al-4V alloy, indicating a good antibacterial effect. It is well known that Zn and Ag ions possess excellent antibacterial functions, and especially Ag has been used as an effective antimicrobial agent incorporated into all kinds of biomaterials, such as metals, polymers, ceramics, and glasses [44,45]. The exact mechanism of Zn and Ag action on bacteria has not been completely understood, but may include interference with electron transport binding to DNA and interaction with the membrane [44,46]. Hu et al. [47] reported that Zn-incorporating TiO_2_ coatings on titanium, generated by plasma electrolytic oxidation, could inhibit the growth of both *Staphylococcus aureus* (*S. aureus*) and *Escherichia coli* (*E. coli*) to a great extent. Moreover, the antibacterial effects of Ag ions in biodegradable Mg-Ag alloys have also been studied. Increased Ag addition (from 2–6%) showed a 74–79% reduction of bacterial viability and a 50–75% reduction of adherent bacteria [18]. Therefore, the novel developed Zn-4Ag alloy could prevent, or at least diminish, postoperative infection.

## 4. Conclusions

A Zn-4Ag alloy was developed as a novel biodegradable Zn-based alloy, and thermal treatment was applied to improve its mechanical properties and to refine the microstructure. The in vitro degradation behavior, cytotoxicity, and antibacterial evaluation were also investigated. Based on the limitations of the in vitro study, the following conclusions can be drawn:

1. After thermomechanical treatment, the yield strength (YS), ultimate tensile strength (UTS) and elongation of the alloy are 157 MPa, 261 MPa, and 37%, respectively, rendering this alloy a promising material for bioresorbable stents. Future alloy development will focus on the optimization of the microstructure to ensure a safe application.
2. The corrosion rate of Zn-4Ag calculated from the released Zn ions in DMEM extract was approximately 10.75 ± 0.16 μg cm^–2^ day^–1^, which is higher than that of pure Zn.
3. A cytotoxic effect decreasing viability and proliferation of L929 and Saos-2 cells was observed, but only in the undiluted extracts of the Zn-4Ag alloy. However, this finding should not be overestimated, since the suitability of the used ISO 10993-5 standard method has to be discussed for degradable materials, according to each application.
4. In vitro antibacterial evaluation showed the Zn-4Ag alloy has the potential to inhibit initial *S. gordonii* adhesion.

Therefore, the biodegradable Zn-4Ag alloy exhibits excellent mechanical properties, predictable degradation behavior, acceptable cytotoxicity, and effective antibacterial property in vitro, which make it a promising candidate for biodegradable implants. It should be investigated by further in vitro and in vivo studies.

## Figures and Tables

**Table 1:** Assessment of mechanical properties by tensile testing and Vickers hardness tests.

Alloy/Processing	Mechanical Properties				References
	Yield Strength (YS_0.2_) (MPa)	Ultimate Tensile Strength (UTS) (MPa)	Elongation to Failure (%)	Hardness (HV1)	
Zn-4Ag *	157	261	37	73	In this study
Zn-4Ag **	149	215	24	82	In this study
WE43/extruded	195	280	10	-	[28]
Zn/cast	10	18	0.32	38	[2]
Zn/extruded	35	60	3.5	-	[2]
Zn/hot rolled	30–110	50–140	5.8–36	39	[2]
Zn-2.5Ag/extruded	147	203	35	-	[17,29]
Zn-5Ag/extruded	205	253	36	-	[17,29]
Zn-7Ag/extruded	236	287	32	-	[17]

* Thermomechanical treatment; ** Additional precipitation hardening. While the aspired precipitation hardening essentially resulted only in a slight increase in hardness, yield strength and ultimate tensile tended to decrease. This may be because precipitates had formed beforehand

**Table 2:** Composition of the blood plasma, DMEM and McCoy’s 5A [30,31,32].

	Inorganic Ions (mmol L^−1^)								Organic Components			Concentrations of Buffering Agents (mmol L^−1^)
Composition Title	Na	K	Mg	Cl	Ca	HPO_4_	SO_4_	HCO_3_	Protein (g L^−1^)	Glucose (mmol L^−1^)	Amino Acids (g L^−1^)	
Blood plasma	142	5.0	1.5	103.0	2.5	1.0	0.5	27.0	63–80	3.6–5.2	Variable	43.5–45.5
DMEM	127.3	5.3	0.8	90.8	1.8	0.9	0.8	44.1	-	4.5	1.6	70
McCoy’s 5A	141.0	5.4	0.8	117.2	1.2	4.2	0.8	26.2	-	16.6	0.4	30.4

**Table 3:** The mean Zn ion concentration in pure Zn and Zn-4Ag alloy extracts.

Cell Medium	Samples	Zn Ion Concentration (μmol/L)			
		100% Extracts	33.3% Extracts	16.7% Extracts	10% Extracts
DMEM	Pure Zn	314.4	107.4	55.5	34.7
	Zn-4Ag	493.4	167.2	85.4	52.6
McCoy’s 5A	Pure Zn	132.8	51.5	31.1	22.9
	Zn-4Ag	174.4	65.4	38.0	27.0
