# Correction: Li, P. et al. Mechanical Characteristics, In Vitro Degradation, Cytotoxicity, and Antibacterial Evaluation of Zn-4.0Ag Alloy as a Biodegradable Material. *Int. J. Mol. Sci.* 2018, *19*, 755

**DOI:** 10.3390/ijms21062258

**Published:** 2020-03-24

**Authors:** Ping Li, Christine Schille, Ernst Schweizer, Frank Rupp, Alexander Heiss, Claudia Legner, Ulrich E. Klotz, Jürgen Geis-Gerstorfer, Lutz Scheideler

**Affiliations:** 1 Section Medical Materials Science and Technology, University Hospital Tübingen, Osianderstrasse 2–8, 72076 Tübingen, Germany; pingli1015@gmail.com (P.L.); Christine.Schille@med.uni-tuebingen.de (C.S.); Ernst.Schweizer@med.uni-tuebingen.de (E.S.); Frank.Rupp@med.uni-tuebingen.de (F.R.); Juergen.Geis-Gerstorfer@med.uni-tuebingen.de (J.G.-G.); 2 Research Institute for Precious Metals and Metals Chemistry (fem), Katharinenstrasse 17, 73525 Schwäbisch Gmünd, Germany; heiss@fem-online.de (A.H.); legner@fem-online.de (C.L.); klotz@fem-online.de (U.E.K.)

The authors wish to make the following corrections to this paper [1]:

The authors have found one inadvertent error in our abstract [1]. In this abstract, “The corrosion rate of Zn-4Ag calculated from released Zn ions in DMEM extracts is approximately 0.75 ± 0.16 μg cm^−2^ day^−1^” should be “approximately 10.75 ± 0.16 μg cm^−2^ day^−1^”.

The authors would like to apologize for any inconvenience caused to the readers by this change.
